# EMIRA: Ecologic Malaria Reduction for Africa – innovative tools for integrated malaria control

**DOI:** 10.3402/gha.v7.25908

**Published:** 2014-11-05

**Authors:** Peter Dambach, Issouf Traoré, Norbert Becker, Achim Kaiser, Ali Sié, Rainer Sauerborn

**Affiliations:** 1 Institute of Public Health, University of Heidelberg, Heidelberg, Germany; 2 Centre de Recherche en Santé de Nouna, Nouna, Burkina Faso; 3 German Mosquito Control Association (KABS), Speyer, Germany; 4 Centre for Organismal Studies, University of Heidelberg Heidelberg, Germany

**Keywords:** Malaria, *Bacillus thuringiensis israelensis*, vector control, study design, larval source management, Burkina Faso

## Abstract

**Background:**

Malaria control is based on early treatment of cases and on vector control. The current measures for malaria vector control in Africa are mainly based on long-lasting insecticide treated nets (LLINs) and to a much smaller extent on indoor residual spraying (IRS). A third pillar in the fight against the malaria vector, larval source management (LSM), has virtually not been used in Africa since the ban of DDT in the 1960s. Within the light of recent WHO recommendations for *Bacillus thuringiensis israelensis* (*Bti*) use against malaria and other vector species, larval source management could see a revival in the upcoming years. In this project we analyze the ecologic and health impacts as well as the cost effectiveness of larval source management under different larviciding scenarios in a health district in Burkina Faso.

**Methods:**

The project is designed as prospective intervention study with duration of three years (2013–2015). Its spatial scale includes three arms of interventions and control, comprising a total of 127 villages and the district capital Nouna in the extended HDSS (Health Demographic Surveillance System) of the Kossi province. Baseline data on mosquito abundance, parasitemia in U5 children, and malaria related morbidity and mortality are gathered over the project duration. Besides the outcome on ecologic and health parameters, the economic costs are seized and valued against the achieved health benefits.

**Conclusions:**

Risk map based, guided larvicide application might be a possibility to further decrease economic cost of LSM and facilitate its faster incorporation to integrated malaria control programs. Given the limited resources in many malaria endemic countries, it is of utmost importance to relate the costs of novel strategies for malaria prevention to their effect on the burden of the disease. Occurring costs and the impact on the health situation will be made comparable to other, existing intervention strategies, allowing stakeholders and policymakers decision making.

Malaria is causing severe health and economic damage in Africa. An estimated 1.2 billion people worldwide were at high risk in 2012 (>1 case per 1,000 population), living mostly in the African Region (47%) and the Southeast Asia Region (37%) (1). An estimated total of 207 million cases in 2012 occurred worldwide, mainly in Africa. Malaria was responsible for an estimated 627,000 deaths in 2012, of which 80% occurred in sub-Saharan Africa. Malaria epidemics tend to occur in regions where endemicity is relatively low and acquired immunities do not last long enough until the next epidemic arises (2). Transmission takes place in particular in rural areas, where vector densities and entomologic inoculation rates are high, due to high vector productivity and a low population density (3). The highest prevalence of cases, in particular deaths, occur among children under the age of five (90%), representing the leading cause of child mortality in Africa.

Since the role of the *Anopheles* mosquito in malaria transmission was discovered more than 100 years ago, different control and eradication attempts have been undertaken. Besides adult vector control and exposure reduction, several strategies are based on vector larvae reduction such as habitat drainage or by targeting the vector larvae directly via insecticides or microbials. A promising approach that came up some 30 years ago is the use of bacteria-produced toxins such as *Bacillus sphaericus* (*Bs*) and *Bacillus thuringiensis israelensis* (*Bti*) that, if digested, kill the larval stages of vulnerable mosquito species (4, 5). Other insects, mammals, and other non-target organisms are not affected. These microbials have proven their efficacy against various mosquito species (6–10) and show an effect on vector populations between 1 and 2 weeks (7, 11–14). The success and cost-effectiveness of microbial-based vector control strategies is strongly dependent on the type and number of larval habitats to which it is applied. Habitat occurrence and types differ by geographic region due to climate, vegetation, and cultural practice and show differences between rural and urban settings.

## Rationale

Even now, larviciding programs still carry the image of being expensive and complicated to run. Limitations of use exist for particular environments (11, 15), for a plurality of settings though, environmental larviciding seems to be a promising approach. There is a multitude of fields of application and its importance is underlined by its recent incorporation in the Roll Back Malaria Partnership of the World Health Organization (1, 16). Although in Western countries, large-scale larviciding programs are in place for more than 30 years (17), in Africa to date this method is used only to a very small extent, mostly in research settings. The general feasibility and effectiveness for specific regions is pointed out by several recent studies that show a remarkable impact of larviciding combined with other interventions on the malaria epidemiology (18–20).

An approach elaborated within the project “Ecologic Malaria Reduction for Africa” (EMIRA) aims on complementing the commonly used integrated malaria control measures (Insecticide Treated Nets (ITNs), DDT residual spraying in houses against resting mosquitoes on walls, intermittent preventive treatment in pregnancy (IPTp), early diagnosis and treatment of malaria cases, and seasonal malaria chemoprevention) by treatment of mosquito breeding grounds with biological larvicides namely *Bacillus thuringiensis israelensis* (*Bti*) (VectoBac^®^).

These known advantages and possibilities of *Bti* based LSM, namely the treatment of habitats where larvae are immobile and concentrated, we try to proof feasible and efficient in the typical setting of a rural, sub Saharan area. We expect to reduce malaria transmission by decreasing vector abundance and achieve an impact on the malaria-induced morbidity and mortality. Furthermore, we hope to generate evidence that the incorporation of novel approaches, such as selective larviciding of the most productive breeding sites guided by remote-sensing-derived risk maps, can contribute to lower economic costs and hence increase opportunities for their use.

### Research gaps

1. Lack of implementation of integrated malaria vector control strategies in sub-Saharan Africa
2. Insufficient data on the additional health benefit of *Bti*-based LSM in rural environments
3. Lack of reliable data on implementation costs of LSM in rural sub-Saharan Africa allowing per capita cost comparison with measures that are already in place (e.g. impregnated bed nets)
4. Lack of implementation and testing of spatio-temporal malaria risk models in actual intervention measures

### Research hypotheses

1. It is feasible to run a *Bti*-based larviciding intervention in a complete health district in a developing country.
2. The incurring costs per capita will be similar to those for the distribution of ITNs.
3. There will be a significant health benefit to reflect in several outcome indicators.
4. The use of remote sensing for selective larviciding in the most productive breeding sites can further cut back on intervention costs.

### Research objectives

Assess the impact of *Bti*-based LSM on malaria vector densities, the transmission of malaria and burden of the disease. Evaluate the cost-effectiveness of satellite-based risk maps for a guided treatment of vector breeding sites (Fig. 2).

## Study design

### Study area

**Fig. 1:**
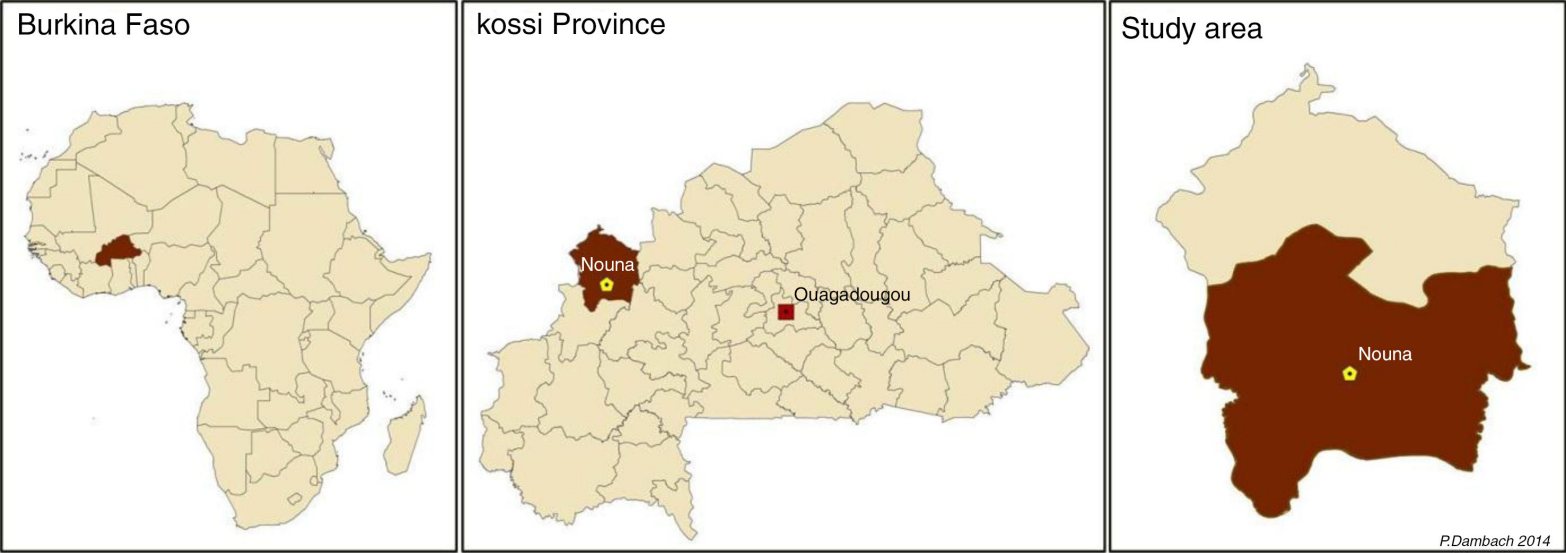
Location of Burkina Faso in Africa, the Kossi Province in North-Western Burkina Faso, and Kossi Province with the study area in its southern part (dark red).

The study is conducted in the rural health district in and around the semi-urban town of Nouna, the capital of the Kossi province, located in the northwestern part of Burkina Faso close to the Mali border at a latitude of 12.73° North and a longitude of 3.86° West (Fig. 1). The city with its approximately 25,000 inhabitants is equipped with major administrative facilities, a hospital, and a research center (21). The population size of the 127 rural villages within the health district ranges from several hundred to a maximum of a few thousand inhabitants. The population density reaches 31 persons per km2 in the rural surrounding. Malaria transmission occurs throughout the year, with a seasonal peak during the late rainy season. The Entomological Inoculation Rate (EIR), which is defined as the number of infective bites per person per year, is reported as high as several hundred (22–24). Within the study region, ITNs are used, and IPT, early diagnosis, and treatment of malaria cases are performed. Indoor residual spraying is absent in the whole study region. The principal malaria vector in the study area is *Anopheles gambiae s.l*., with more than 90% followed by *A. funestus* and *A. nili*. Typical larval breeding sites appear in a variety of shapes. Common are pools and ponds that serve as water holes for cattle. Brickworks and clay pits are anthropogenic water bodies in the direct vicinity of villages. Those are mostly few and well limited, hence ideal terrain for larviciding. The distribution of other breeding sites such as wet-rice fields and submerged areas is heterogeneous and restricted to few areas. Only approximately 6% of villages feature these types of breeding sites. Breeding site ecology is different and larval densities were found to depend on the availability of vegetation, nutrients, and water quality (25).

A main reason for undertaking this research program within the greater Nouna region is that about half of the total population of ~155,000 within the study region is part of a Health Demographic Surveillance System (HDSS) (21). Routinely collected data on causes of death and morbidity facilitate the Project's impact evaluation regarding malaria transmission and malaria-related deaths.

### Implementation of LSM measures

The project is designed as a prospective intervention study with a duration of 3 years (2013–2015). Its spatial scale includes three arms of interventions and control, comprising a total of 127 villages and the district capital Nouna in the extended HDSS of the Kossi province (21). Baseline data were gathered in 2013 while the larviciding intervention runs in 2014 and 2015 accompanied by the continuous collection of impact evaluation data.

**Fig. 2:**
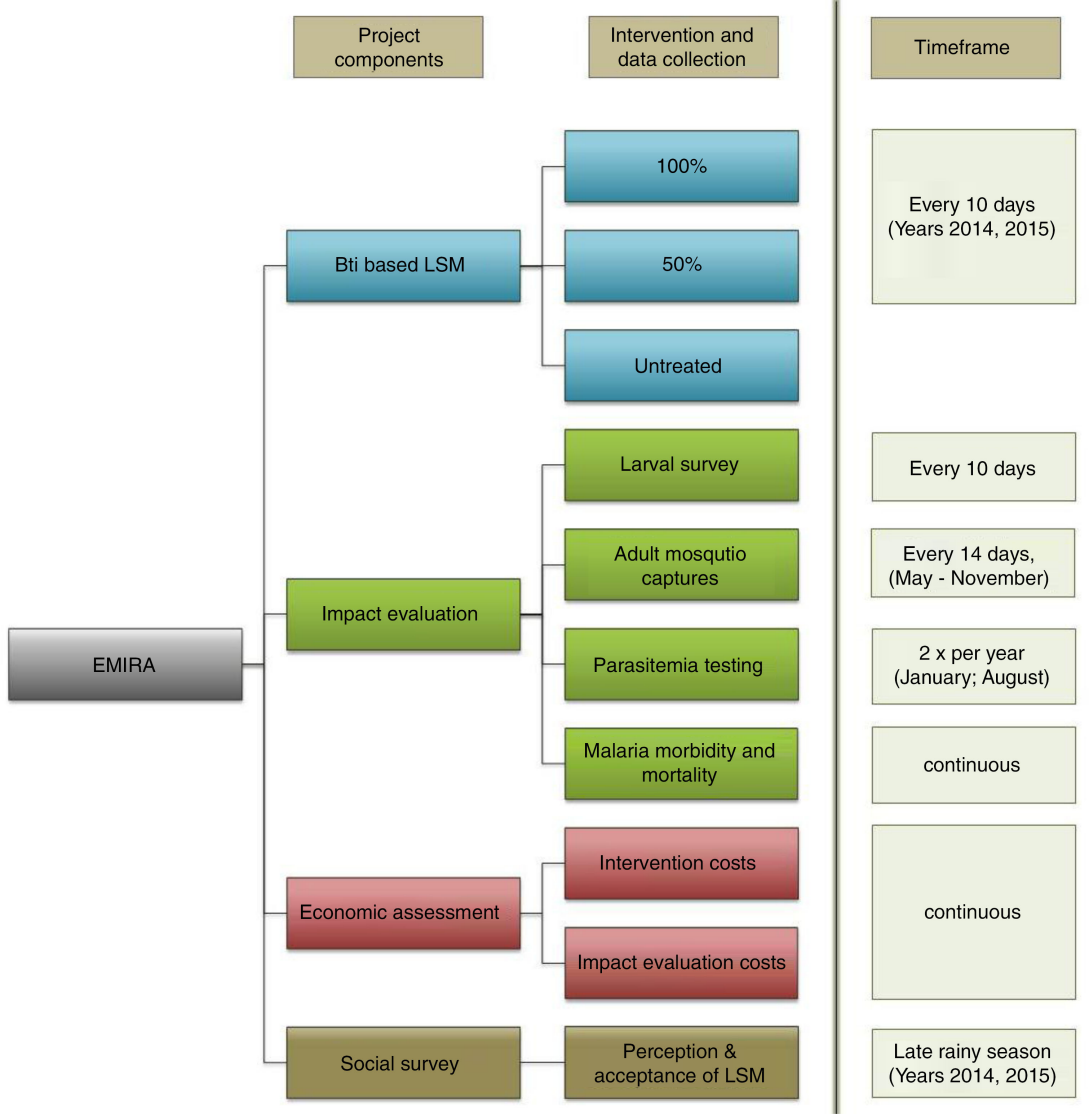
Work Breakdown Structure of the EMIRA Project. Project components, intervention and data collection, and the timeframe during which actions are performed are shown. The project components ‘*Bti*-based LSM’ and ‘Social’ are only run during years with larviciding (2014, 2015).

The three arms are distinguished by their level of intervention as follows:

1. Exhaustive treatment of all detected breeding sites in and around 42 villages and the semi-urban district capital Nouna during the rainy season (extended for prevailing breeding sites). Larvicide application is performed using detailed maps of the villages and knowledge of the terrain.
2. Guided treatment using remote-sensing-derived risk maps for 42 villages. The 50% of most productive breeding sites regarding their larval densities are treated selectively. Maps for steered treatment are based on a prediction model using high spatial resolution satellite imagery (25, 26). A 50% threshold for treatment was chosen under cost-effectiveness aspects, reflecting a considerable retrenchment of costs and simultaneously being expected to still have a noticeable impact on reducing malaria transmission by cutting on vector abundance.
3. Control arm with no performed larval control for 43 villages but collection of impact evaluation parameters (Fig. 2).

The mosquito breeding sites are treated with *Bti* every 10 days to reduce the number of emerging adult vectors. The larviciding runs as an added component to complement the integrated malaria control that is uniformly implemented in the study region (use of ITNs, IPTp, early diagnosis, and treatment of malaria cases). During the rainy season (June-September), local village-based teams perform the larvicide application and the ground surveillance of its efficacy. Teams of sprayers and entomologic technicians treat the breeding sites in a repetition cycle of 10 days. Existing infrastructures of other anti-malaria measures such as bed net distribution and basic medical treatment on the district level (rural health centers, administrative structures) facilitate an implementation and integration of this method. Information on proximity of houses to breeding sites is available via GIS databases that comprise the position of breeding sites and the geo-coordinates of all households within the HDSS area.

### Clustering of villages

**Fig. 3:**
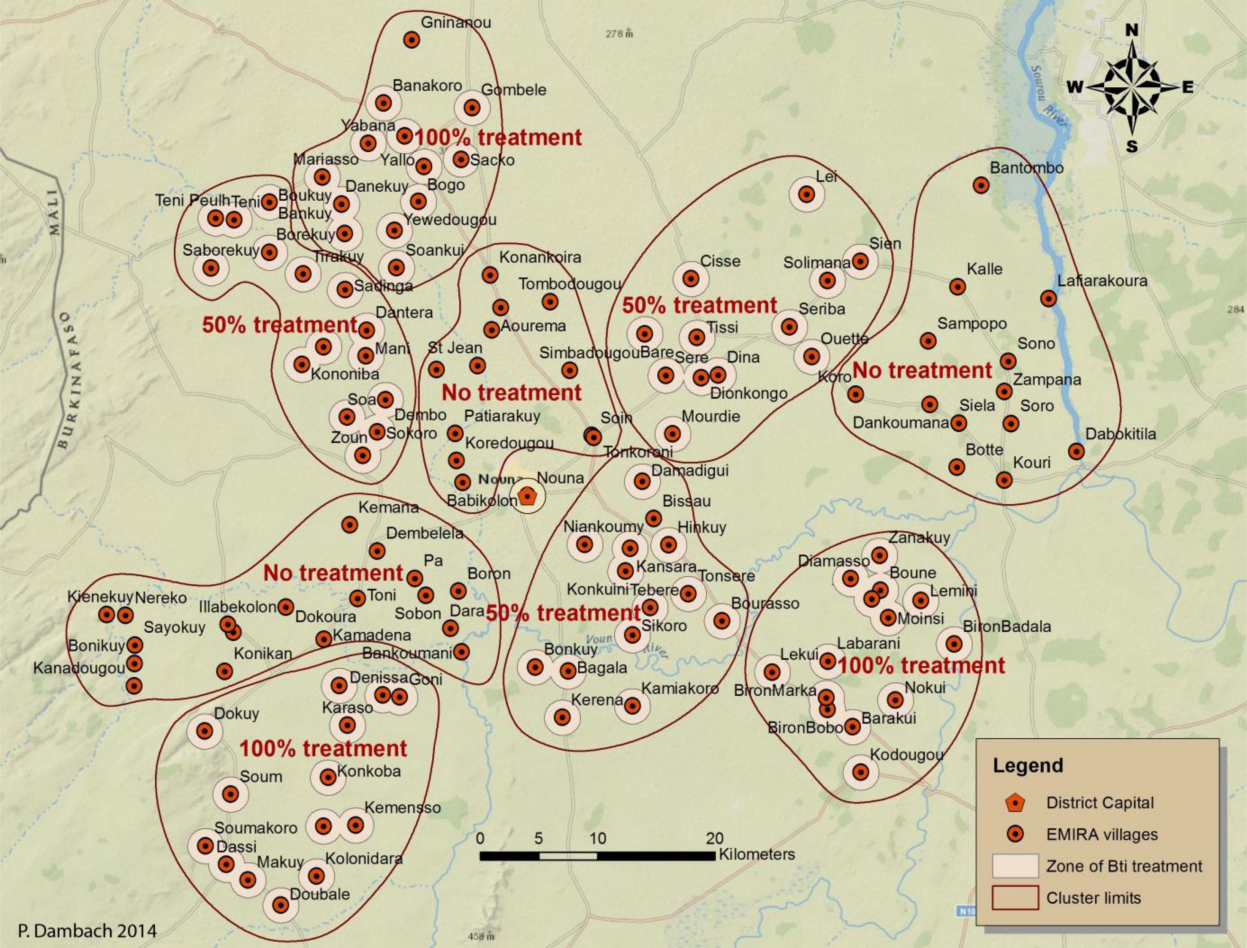
Clustering of study villages according to their proximity to each other, minimizing contamination effects of neighboring clusters. Therefore, clusters may contain different numbers of villages and population. Zones of intervention (100% treatment, 50% guided treatment) are shown as dissolved buffers. Buffers are drawn 1 km around approximate village outline, taking into account the average vector mosquito flight range. Other protective measures against malaria that are already in place (e.g. impregnated bed nets) were not modified and can be assumed to be equally distributed within the study region.

All 127 rural study villages are regrouped into a total of nine clusters (Fig. 3). These are based on coherent, interconnected areas of village buffers (proximity), covering a similar number of villages. Each study arm is split into three clusters with identical intervention or control, respectively. This approach minimizes confounding factors that may arise from different natural factors such as topography, hydrology, and climate. Regional differences in ecology are addressed in the sense that intervention types of each study arm are represented in each type of ecologic environment (proximity to riverine system, more arid areas). These also reflect the differences in the performed type of agriculture (e.g. wet-rice cultivation, crops adapted to aridity). Larviciding is performed within a buffer of 1.5 km around the village outline, which will minimize mosquito intrusion from the outskirts of the village. An additional protective effect for treated areas is achieved by clustering villages according to their proximity to each other; this means villages of one cluster can have overlapping buffer zones, and villages from different clusters cannot.

### Remote sensing and risk maps

The risk of pools and surface water accumulation for their productivity of malaria vector mosquitoes can be assessed by prediction models that were trained with ground data (25–27). Those models are based on the identification of environmental factors that are detected via satellite imagery. The most important prediction parameters are distinguishable water parameters, for example, water quality, presence of phyto-/zooplankton, vegetation, color (28, 29). Amending parameters are land temperature, rainfall, and NDVI (Normalized Difference Vegetation Index – a combination of satellite image bands to detect vegetation, which is a proxy for surface water) (25, 30, 31). Less organic polluted water and prevalence of phyto-/zooplankton is important for *Anopheles* larval growth, while pollution, strong turbidity, and complete vegetation cover is a hindering factor. In the investigation area, there are pools and tarns during the rainy season, which can be of different appropriateness for vector breeding. In research projects prior to EMIRA in Burkina Faso and Senegal, methods to classify breeding sites by their anopheline larval productivity using satellite imagery were developed (25–27, 32). Due to the high spatial resolution of 2.5 m and spectral resolution SPOT 5 (Satellite Pour l'Observation de la Terre) data were used for risk map creation. Within EMIRA, this novel tool will be tested as a method for significantly reducing the use of *Bti* by facilitating a guided treatment of the most productive breeding sites.

### Impact evaluation

#### Data collection

The impact evaluation data are raised over the project duration of 3 years, allowing estimating the intervention's impact on vector densities and human health. Comparability between intervention years and the non-intervention year 2013 pursued as well as the spatial comparability between intervention and control villages grouped in clusters.

The effectiveness of the intervention measures is examined by the following key figures:

1. Vector larvae counts are performed in all treated breeding sites 1 day after larvicide application to ensure correct and sufficient treatment. Larval counts serve as the first and most directly influenced parameter of impact evaluation and assure the efficacy of *Bti* and its correct application in the field.
2. Adult mosquitoes are captured using light traps (Model: Center for Disease Control, Gainesville, Florida) that are positioned in four villages in each cluster. In each village, three sample points are chosen with one outdoor and one indoor light trap each. Data collection is performed every 14 days from May to November and collected mosquitoes are determined to species level.
3. Measuring parasitemia in children aged less than 5 years: Two rounds of testing for malaria parasites are performed every year. Rapid Diagnostic Test (RDT) sets are used in children with increased temperature to test for malaria. For positive cases, a microscopy test is performed in the district hospital to determine blood parasite rates. The activity is carried out once before the onset of the rainy season and once during the time of highest infection, in August. A total of 19,700 children under five live within the total population, and sampling is performed in the calculated sample size of 385 children.
4. Cases of death and morbidity reported by the HDSS and additional Community Key Informants (CKI). The HDSS data allow comparing death cases and morbidity attributable to malaria between study years and between treated and untreated villages in the same year. The HDSS data are provided on a regular basis three times per year. Morbidity cases are collected from the consultation registers in the health facilities since origins of patients are not available from the database of the health district.

### Assessment of costs

The registration of incurred costs for baseline data collection and LSM implementation is conducted alongside the study. This study analyzes two different scenarios, full and guided partial larviciding, and compares those against each other. The analysis for full and guided intervention captures all resources used, comprising material, transport, and costs for scientific and field personnel. Furthermore, the inclusion of opportunity costs allows attribution of available means (e.g. staff time, equipment, and infrastructure). The overall cost for running the project in a research setting will be equally captured as the pure LSM implementation costs. Assessment of the latter is important to calculate per capita cost for LSM and to compare its resource exigencies to other interventions such as impregnated bed nets. Cost efficiency of full and guided LSM will be evaluated by comparing with the registered health outcome parameters.

### Perception and acceptability

The LSM is accompanied by a qualitative study component researching the perception and acceptability of LSM within the population. The perceived reduction in mosquito abundance and nuisance will be compared with the number of mosquitoes caught with light traps. Furthermore, the general acceptance and will of participation in a larviciding program will be registered to improve information and participation strategies.

## Conclusions

Malaria as the leading cause of death in wide parts of sub-Saharan Africa is in the scope of the global health priorities. EMIRA's cooperative and multidisciplinary approach is designed to evaluate the feasibility, added health benefit, and cost-effectiveness of novel tools for larval source management in these environments. With the current success of ITN programs in many parts of Africa, more and more areas that have previously been highly burdened with malaria, now show characteristics of low and focal malaria transmission and hence offer great opportunities for the implementation of LSM. Risk map-based, guided larvicide application might be a possibility to further decrease economic cost of LSM and facilitate its faster incorporation to integrated malaria control programs. Given the limited resources in many malaria endemic countries, it is of utmost importance to relate the costs of novel strategies for malaria prevention to their effect on the burden of the disease. Occurring costs and the impact on the health situation will be made comparable to other, existing intervention strategies, allowing stakeholders and policymakers decision-making. The project results, in particular the costs incurred and the expected positive effect on the health situation, are monitored and remitted to the Ministry of Health. Based on the outcomes of this project, the national authorities may solicit funds from other donors for the long-term implementation and scale-up for public policies in a later step.
